# Cardiovascular System in COVID-19: Simply a Viewer or a Leading Actor?

**DOI:** 10.3390/life10090165

**Published:** 2020-08-27

**Authors:** Filiberto Fausto Mottola, Nicoletta Verde, Riccardo Ricciolino, Marco Di Mauro, Marco Giuseppe Migliaccio, Vincenzo Carfora, Giorgio Spiniello, Nicola Coppola, Vanvitelli COVID-19 Group

**Affiliations:** 1 Department of Translational Medical Sciences, University of Campania “Luigi Vanvitelli”, 80131 Naples, Italy; filiberto-mottola@hotmail.it (F.F.M.); nicolettaverde1990@gmail.com (N.V.); riccardo.ricciolo91@gmail.com (R.R.); marco.dimauro4@gmail.com (M.D.M.); marcog.migliaccio@gmail.com (M.G.M.); vincenzo.carfora2@gmail.com (V.C.); giorgio.spiniello@gmail.com (G.S.); 2 Department of Mental Health and Public Medicine, Infectious Diseases Unit., University of Campania “Luigi Vanvitelli”, 80131 Naples, Italy

**Keywords:** SARS-CoV-2 infection, cardiovascular system, myocardial injury, heart failure

## Abstract

As of January 2020, a new pandemic has spread from Wuhan and caused thousands of deaths worldwide. Several studies have observed a relationship between coronavirus disease (COVID-19) infection and the cardiovascular system with the appearance of myocardial damage, myocarditis, pericarditis, heart failure and various arrhythmic manifestations, as well as an increase in thromboembolic risk. Cardiovascular manifestations have been highlighted especially in older and more fragile patients and in those with multiple cardiovascular risk factors such as cancer, diabetes, obesity and hypertension. In this review, we will examine the cardiac involvement associated with SARS-CoV-2 infection, focusing on the pathophysiological mechanism underlying manifestations and their clinical implication, taking into account the main scientific papers published to date.

## 1. Introduction

Since December 2019, a new zoonotic betacoronavirus (SARS-CoV-2) has spread all over the world from Wuhan in China [1], known as coronavirus disease (COVID-19). By 16 August 2020, about 21.2 million cases were recorded with about 761,000 deaths worldwide. It is an RNA virus, identified as belonging to the Betacoronavirus family, which also includes MERS-CoV, identified in 2012 as the cause of the Middle East respiratory syndrome, and SARS-CoV, identified in 2002 and responsible for severe acute respiratory syndrome (SARS) [1].

The incubation period varies from 1 to 14 days, although cases with longer incubation periods of up to 24 days have been identified. SARS-CoV-2 infection may cause a wide range of clinical presentations, from an asymptomatic to a severe forms. The main clinical symptoms develop within about 10 days and include dry cough, fever, fatigue, dysgeusia and anosmia; other less characteristic symptoms are headache, runny nose, sore throat, nasal congestion, poor appetite and diarrhea [2,3].

Even though the mortality rate in China was approximately 2%, for reasons still not clear, in some geographical areas (Italy, Spain, USA, UK) it is significantly higher. A possible reason may be a more advanced age, the presence of co-morbidities, differences in the healthcare system, suddenly overcrowded healthcare systems and differences in social distancing [4]. Several studies have shown that numerous risk factors are associated with a worse outcome in patients with SARS-CoV-2 infection. Older age, cancer, diabetes mellitus, hypertension, obesity and previous heart disease are associated with an increased risk of severe forms and mortality [5,6,7,8]. When serious and non-serious patients were compared, the odds ratios (ORs) of hypertension, respiratory system disease and cardiovascular disease (CVD) were 2.36 (95% confidence interval (CI): 1.46–3.83), 2.46 (95% CI: 1.76–3.44) and 3.42 (95% CI: 1.88–6.22), respectively [9]. COVID-19 causes cardiovascular complications, including diffuse thrombosis, pulmonary thromboembolism, disseminated intravascular coagulation (DIC), myocarditis, pericardial effusion, both hypokinetic and hyperkinetic arrhythmias, but also cardiogenic shock. In addition, drugs currently in use for the treatment of COVID-19, such as hydroxychloroquine, azithromycin and protease inhibitors, can affect the cardiac conduction system leading to an extension of the QT interval, which in turn can predispose the onset of ventricular arrhythmias, in particular torsades de pointes.

This review will examine the cardiovascular involvement, direct and indirect, associated with SARS CoV-2 infection in order to manage the cardiovascular complications in the clinical practice.

## 2. Methods

A comprehensive computerized literature research was made to identify studies analyzing the cardiovascular implication of COVID-19 patients using MEDLINE, Google Scholar and EMBASE from December 2020 up to 25 May 2020, involving both medical subject heading (MeSH) terminology and relevant keywords for search strings to locate articles. The following items were used: “cardiovascular complications”, “heart”, “cardiac involvement”, “COVID-19” and “SARS-CoV-2”. The references of studies or reports were checked to find relevant information.

## 3. Cardiac Involvement in Patients with COVID-19

**Figure 1:**
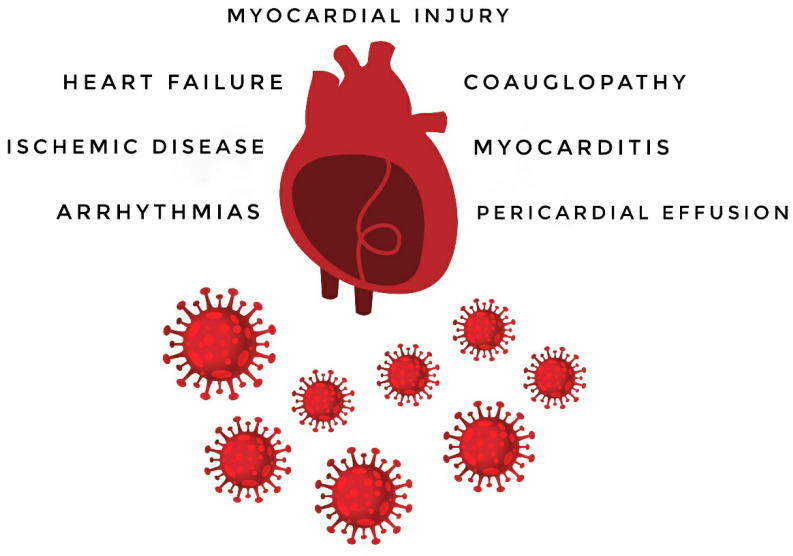
SARS-CoV-2 and cardiovascular involvement.

In COVID-19 patients, myocardial injury, myocarditis, pericardial effusion, new onset or worsening of a previous heart failure, arrhythmic complications and an increased thromboembolic risk have been observed. Figure 1 and Table 1 summarize the cardiovascular involvement in SARS-CoV-2 infection.

### 3.1. Myocardial Injury

An increase in myocardial necrosis indices, such as troponin (TnT), the creatine kinase–myocardial band test (CK-MB) and myoglobin, has been reported in several patients with COVID-19. Wang et al. in a single-center study showed that 7.2% of 138 hospitalized patients developed acute heart damage and that the patients who received care in the intensive care unit (ICU) were more likely to have heart damage (22.2%) compared to non-ICU patients [7].

Shi et al. observed at presentation a high value of TnT in approximately 20% of 416 hospitalized patients with COVID-19. Compared to those without an increase in TnT, these patients were more likely to require invasive or non-invasive ventilation (22% vs. 4%, and 46% vs. 4%, respectively) and to develop acute respiratory distress syndrome (59% vs. 15%) or acute kidney injury (9% vs. 0%; *p* < 0.001 for all); in addition, the mortality rate was higher (51.2% vs. 4.5%; *p* < 0.001) and it increased with the entity of the reference value of high-sensitivity troponin I (hs-TNI) [8].

A recent meta-analysis showed that cardiac troponin I (cTnI) values were significantly higher in patients with severe SARS-CoV-2 infection compared to those observed with mild forms [14].

Guo found that compared with patients with normal TnT levels those with higher levels had significantly higher levels of other heart damage biomarkers, specifically CK-MB and myoglobin, and also had higher levels of N-terminal pro–brain natriuretic peptide (NT-proBNP). Finally, they had a significantly higher level of high-sensitivity C-reactive protein, D-dimer and procalcitonin. During hospitalization, the patients with elevated TnT levels developed more frequent complications, including acute respiratory distress syndrome, malignant arrhythmias, acute coagulopathy and acute kidney damage [15].

The pathophysiological mechanisms underlying myocardial injury caused by COVID-19 are not well defined. An increase in the cardiac necrosis index may be caused by an increased oxygen demand by the myocardium, similar to what happens during a type 2 myocardial infarction, or due to an inflammatory process caused by an exaggerated cytokine response by type 1 and 2 helper T cells, which could cause instability of the atherosclerotic plaque. Huang et al. found that the patients with COVID-19 admitted to an ICU had higher serum cytokine levels, including interleukin IL-2, IL-7, IL-10, granulocyte colony stimulating factor, and tumor necrosis factor α. The activation or advanced release of these inflammatory cytokines leads to apoptosis or necrosis of myocardial cells, also leading to an increase in the indices of myocardial necrosis [16]. Another explanation may be direct damage of cardiomyocytes by the virus [15,17]. Different studies speculate that the interaction between SARS-CoV-2 and angiotensin-converting enzyme 2 (ACE-2) in the heart could contribute to SARS-mediated myocardial inflammation and damage [8,15,18].

Concluding on this point, it should be stressed that myocardial necrosis index measurements were likely to have been performed in those who were most ill or where there was reasonable suspicion of myocardial ischemia or myocardial dysfunction.

### 3.2. Myocarditis and Pericardial Effusion

A case of myocarditis in a patient with MERS-CoV infection was found to have acute heart failure, documented also by cardiac magnetic resonance imaging (MRI) [19]. Similarly, in some patients with or without pre-existing CVD, COVID-19 was associated with myocarditis.

Incardi et al. and Fried et al. published clinical cases of patients with chest pain and diffuse ST increase with elevated troponin and negative coronary angiography, but who subsequently resulted positive for COVID-19; myocarditis was later confirmed with cardiac MRI in both cases [20,21].

Pericardial involvement has not been well described, although in our experience, we have shown cases with COVID-19 and concomitant pericardial effusion (personal data not shown), one of which about a week after discharge in the absence of another triggering cause. Indeed, several patients have been shown to have a feeling of chest tightness similar to that of an acute coronary syndrome in the absence of coronary obstruction and in the absence of an increase in myocardial necrosis indices and therefore attributable to the symptoms of COVID-19 [22]. Further studies are needed to better clarify this possible association.

### 3.3. Heart Failure

In other infections (influenza, other coronaviruses, and others), patients with CVD have been shown to have an increased risk of heart failure during acute infections [23,24].

Furthermore, in COVID-19 patients a worsening in underlying heart failure has been observed [25]. In a retrospective cohort study on 191 patients, Zhou et al. showed that with cardiac complications, such as a worsening of a previous heart failure or a new onset, myocardial infarction was common in patients with pneumonia, with cardiac arrest present in approximately 3% of patients; predisposing risk factors were the severity of pneumonia, advanced age and a pre-existing heart disease [26].

Moreover, an increase in N-terminal pro b-type natriuretic peptide (NT-proBNP) has been linked to the severity of inflammation and worsening left ventricular function, and serious increases in NT-proBNP and TnT have been shown in patients with worse outcomes than those who showed favorable outcomes [8,15,17].

In a case series, Chen et al. observed increased levels of NT-proBNP and cTnI in 27.5% and 10% of patients, respectively. Interestingly, levels of IL-6 and other inflammatory cytokines, such as the expression of cytokine storm, were elevated especially in patients who experienced a more severe disease course requiring ICU admission. [27].

A recent Latif study found that the mortality rate on 28 heart transplant (HT) patients with concomitant COVID-19 infection was 25%. The majority (76%) had evidence of myocardial damage and high inflammatory biomarkers. HT patients may be at increased risk of infection and adverse outcomes due to a number of common co-morbidities after heart transplantation, including hypertension, diabetes and cardiac allograft vasculopathy. Furthermore, all require maintenance immunosuppression, which predisposes, on the one hand, to a greater infection risk and, on the other, it can have a protective action against the cytokine storm [28].

However, whether the heart failure is due to a worsening in the left ventricular function in a patient with previous decompensation or is due to a new cardiomyopathy is still to be demonstrated, as well as the repercussions on the right ventricle, especially in patients with severe pulmonary micro-thromboembolism in acute respiratory distress syndrome (ARDS).

### 3.4. Direct Arrhythmic Risk Associated with COVID-19

Few data are available in the literature on the incidence and management of cardiac arrhythmias related to COVID-19.

Wang et al. in 138 patients with COVID-19 pulmonary infection in Wuhan observed cardiac arrhythmias in 23 (16.7%) patients and acute cardiac injury in 10 (7.2%). They reported heart palpitations as one of the most common initial symptoms of the disease (7.3%) [7]; moreover, exacerbation and the new onset of paroxysmal supraventricular tachycardia (PSVT), atrial fibrillation (AF) and flutter were possible in patients with COVID-19. In patients with severe pneumonia, ARDS and sepsis, the incidence of AF during hospitalization was very high (about 23–33% of critically ill patients) [29].

In 355 Italian COVID-19 patients who died, a retrospective chart review identified a history of AF in 24.5% [30]. During hospitalization, malignant ventricular arrhythmias, defined as sustained ventricular tachycardia (VT) or ventricular fibrillation (VF), occurred in 11 (5.9%) patients [22].

Arrhythmias may be due to myocardial damage; in fact, in patients with elevated TnT, a higher incidence of ventricular arrhythmia was reported. Therefore, myocardial injury might result in atrial or ventricular fibrosis, thus, the substrate for subsequent cardiac arrhythmias even after hospital discharge and MRI can help us stratify these patients.

Direct viral infection, hypoxia-induced apoptosis and association with the cytokine storm may be the mechanisms causing arrhythmias. Systemic inflammatory response syndrome can be an important risk factor for arrhythmia onset: IL-6 directly inhibits the human ether-à-go-go-related gene (hERG) K^+^ channel and prolongs action potential duration in ventricular myocytes [31]; indirectly, the systemic inflammatory response hyper-activates the cardiac sympathetic system via central hypothalamus-mediated (inflammatory reflex) and peripheral (left stellate ganglia activation) pathways [32]. However, myocardial damage alone is not enough and there are other factors involved in enhancing the arrhythmic risk in COVID-19: in fact, in these patients, only half showed acute cardiac injury despite the high frequency of arrhythmias [32].

### 3.5. Indirect Arrhythmic Risk Associated with Anti-SARS-CoV-2 Treatment

An important role in the development of arrhythmias may be played by pharmacological treatment used for COVID-19 patients that increases the susceptibility to QT-related life-threatening ventricular arrhythmias, particularly torsades de pointes. In fact, many drugs used to treat these patients have the ability to block cardiac potassium currents, with subsequent prolongation of the QT-interval and an increased risk for arrhythmias [32]. In particular, chloroquine (CQ) and hydroxychloroquine (HCQ) are drugs used for these patients with known QT-prolonging effects [33]. These drugs may increase the depolarization length duration and Purkinje fiber refractory period, leading to atrioventricular nodal and/or His system dysfunction [34,35,36]. In 2015, Capel et al. demonstrated an inhibitory effect of HCQ on the hyperpolarization-activated current ion channels (also known as “funny current” channels) [37]. These findings seems to correlate with a proposed mechanism by which refractory action potentials in cardiac myocytes may lead to a prolongation of the QT interval due to delayed depolarization and repolarization from abnormal ion currents. In an observational study in New York in hospitalized patients with COVID-19, treatment with HCQ, azithromycin, or both, compared with neither treatment, was not significantly associated with in-hospital mortality, but cardiac arrest was significantly more likely in patients receiving HCQ + azithromycin [38]. In another study, about 10% of COVID-19 patients treated with these drugs developed QT prolongation, with ventricular arrhythmia in 2 COVID-19 patients out of a group of 28 treated with high-dose chloroquine [39].

A Chinese meta-analysis including 2613 patients in 11 randomized controlled trials observed that HCQ had an increased risk of mild adverse events compared to placebo CQ. In addition, protease inhibitors (lopinavir/ritonavir; darunavir/ritonavir, darunavir/cobicistat) administrated in patients with COVID-19 inhibited CYP3A4, which may further increase plasma levels of QT-prolonging drugs (in particular macrolides like azithromycin or fluoroquinolones, frequently administered to these patients) [40].

Therefore, some simple guidance could be useful for the management of these patients. While ECG monitoring is always available for COVID-19 patients admitted to ICU, in patients hospitalized in non-intensive rooms, clinicians should monitor QT-intervals according to the risk factors. In patients with congenital or acquired long QT syndrome (LQTS), in those treated with other QT-prolonging drugs and/or with structural heart disease or bradycardia, ECG may be evaluated at baseline, 4 h after administration of CQ or HCQ and/or anti-viral therapy and then every 1–3 days. In all other patients, QT-interval monitoring should be performed 24 h after the start of therapy; if there is a worsening kidney/liver function and electrolyte disorders (in particular K^+^, Ca^2+^ and Mg^2+^), QTc-interval monitoring is indicated.

It is important to pay attention to the particular clinical situation, like diarrhea, which may lead to hypokalemia, which may influence the QTc interval. Moreover, all unnecessary QT prolonging drugs should be stopped. If QTc is higher than 500 ms or if QTc increases by ≥60 ms from baseline, then the safety of QT prolonging antiviral drugs should be reviewed, and serum potassium levels should be kept at >4.5 mEq/L; fever should be aggressively treated with paracetamol.

The treatment of arrhythmia is the same for COVID-19 and non-COVID-19 patients. The treatment goals in all patients with AF must consider ventricular rate control, rhythm control and thromboembolic prophylaxis. We should remember that the combination of amiodarone, the choice of antiarrhythmic medication for rhythm control, with HCQ and/or azithromycin should preferably be avoided. Drug interactions should always be considered before the administration of any drugs. Asynchronous defibrillation should be performed in patients with VF, while synchronized electrical cardioversion should be performed in hemodynamically unstable VT. Lastly, if there is no important drug interaction, we can attempt a pharmacological conversion. In patients with severe acute respiratory insufficiency, the correction of underlying reversible triggers should be considered and could interrupt the arrhythmia [41].

For the prevention of torsade de pointes in the setting of COVID 19 infection, we can withdraw all QT prolonging drugs and normalize the potassium level (target > 4.5 mEq/L), give intravenous magnesium supplementation and finally increase the heart rate by withdrawing bradycardiac agents, and if needed by i.v. isoproterenol or temporary pacemaker.

## 4. Thromboembolic Risk and Vascular Involvement in COVID-19

Vascular involvement in COVID-19 is demonstrated by the high risk of thromboembolism observed in these patients. Different case reports, case series and retrospective studies suggested that the incidence of pulmonary embolism (PE) in patients with COVID-19 infection might be high [42,43].

In Lille University, in a case series on 107 COVID-19 patients admitted to the ICU for pneumonia, the authors observed an unexpected high prevalence (20.6%) of PE; despite a similar severity score at the entrance to the ICU, the frequency of PE in their COVID-19 series was twice as high as the frequency they found in the same time interval in 2019 [10].

An interesting observational study based on 184 patients with COVID-19 pneumonia showed a high prevalence of venous thromboembolism confirmed by CT pulmonary angiogram and/or ultrasonography (27%, 95% CI 17–37%), and of arterial thrombotic events (3.7%, 95% CI 0–8.2%) [11].

In an Italian study enrolling 362 consecutive patients, thromboembolic events occurred in 28 (7.7%): 16 with venous thromboembolism (10 with pulmonary embolism with or without deep vein thrombosis and 6 with isolated deep venous thromboembolism) and the remaining patients with ischemic stroke and/or myocardial infarction [12].

Similar results (23% of PE) were obtained in a retrospective study conducted by French researchers in patients with COVID-19 [13].

The pathogenic mechanism of a high prevalence of thromboembolic events may be linked to the hypercoagulative state observed in COVID-19 patients. COVID-19 has been described as causing a proinflammatory state due to cytokine-mediated diffuse microvascular damage [44,45,46,47]. Some authors observed a close interaction between high D-dimer values and adverse events in COVID-19 [26,48,49]. Indirect suggestions of the hypercoagulative state were the observations of an advantage in the use of thromboprophylaxis in COVID-19 patients. For example, one study observed a mortality benefit from thromboprophylaxis with subcutaneous unfractionated heparin or low molecular weight heparin in 19 COVID-19 patients with highly elevated D-dimer [50].

A higher incidence of acute PE in patients with COVID-19 infection should be suspected when a respiratory worsening, unexplained tachycardia, a fall in hypovolemia or sepsis, ECG changes suggestive of PE, and signs of deep vein thrombosis are observed. Moreover, in these patients, there is an urgent need to improve specific venous thromboembolism (VTE) diagnostic strategies, taking into account some limits: computed tomography pulmonary angiogram (CTPE) is often delayed or not performed due to co-morbid renal failure and cardiopulmonary instability, leading to an unacceptable risk for transfer; similarly, duplex ultrasonography (DUS) is difficult to perform due to the large number of patients and difficulty in completely disinfecting the machines. Thus, it is very important to use diagnostic scoring systems such as the Wells’ criteria, Pulmonary Embolism Rule-out Criteria (PERC), or the Geneva scoring system [51,52,53].

Concluding on this point, trials evaluating the correct management of thrombotic complications in these patients are needed, considering that novel oral anticoagulants (NOACs) such as lopinavir/ritonavir may interact with drugs for COVID-19 and, thus, they should be avoided.

## 5. COVID-19 and Renin–Angiotensin System Inhibitors

Since COVID-19 infects patients through the link to the ACE2 receptor, it has been speculated that the use of angiotensin-converting enzyme inhibitor (ACEI) and an angiotensin receptor blocker (ARB) may contribute to an increase in the level of ACE2, which makes the virus more likely to invade cells. In fact, based on previously conducted studies on SARS-CoV-1, the SARS-CoV-2 virus has been shown to express numerous spikes of protein S on the surface of the viral envelope that were critical for the transmission of infection. The S glycoprotein includes two subunits with different action: S1 influences the virus tropism and attachment to the external membrane, while S2 is responsible for virus cell fusion and effective cell entry. This protein binds to the ACE2 through the S1 subunit; due to the presence of transmine membranes serine proteases 2 (TMPRSS2), also expressed by the host cell, which was able to perform the protein priming, it was essential to allow the virus to enter the cell. After these processes, the virus can enter the sarcoplasmic reticulum and begin its RNA replication [54,55]. ACE-2 is expressed on the surface of a variety of host cells, including type I and type II pneumocytes, pericytes, cardiomyocytes, along with other cells of the digestive system such as enterocytes in the small intestine and finally in arterial and venous endothelial cells [4]. Interestingly, circulating ACE2 levels in patients were gender dependent, and in a study published recently, researchers found that the distribution of ACE2 is more widespread in males than in females. Moreover, different ACE polymorphisms were observed in different races, and this may suggest a possible link with the COVID-19 diffusion and with the different outcome observed throughout the world [5,6]. On the other hand ACE2 also serves a role in lung protection, and different studies show that ACE inhibitors/ARBs may potentiate the lung protective function of ACE by reducing angiotensin II levels, which is pro-inflammatory, pro-thrombotic and pro-oxidant [5,7,8]. Finally, in a recent study, Metha et al. showed no association between ACEI or ARB and a higher probability of positivity to the COVID-19 test [56].

Thus, it should be underlined that these drugs are often used in the treatment of patients with underlying chronic diseases and therefore in more fragile patients who are already at a greater risk of mortality and complications.

Because of the important role played by inhibitors of the renin–angiotensin system (RAS inhibitors) in patients with heart disease, particularly in those with heart failure, and since there is no clinical evidence to support the adverse or beneficial effects of RAS inhibitors in COVID-19 patients with cardiovascular disease, major cardiovascular societies recognize that patients with ACEI or ARB should not stop the treatment.

## 6. Management of Cardiovascular Complications

The management of patients with COVID-19 and cardiovascular complications is not yet well defined.

**Figure 2:**
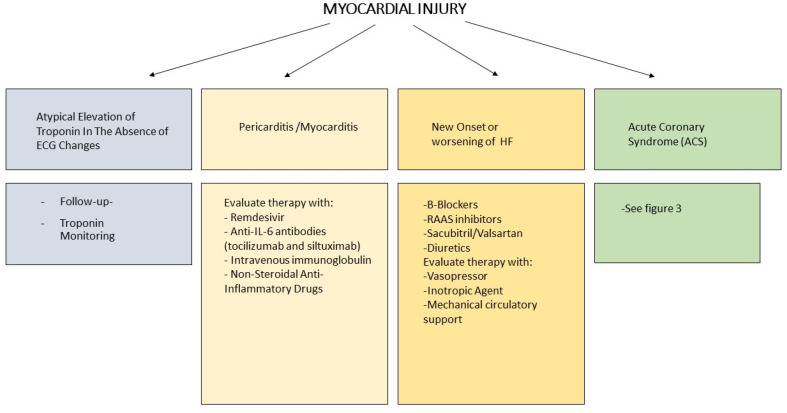
SARS-CoV-2 and management. Heart Failure (HF).

**Figure 3:**
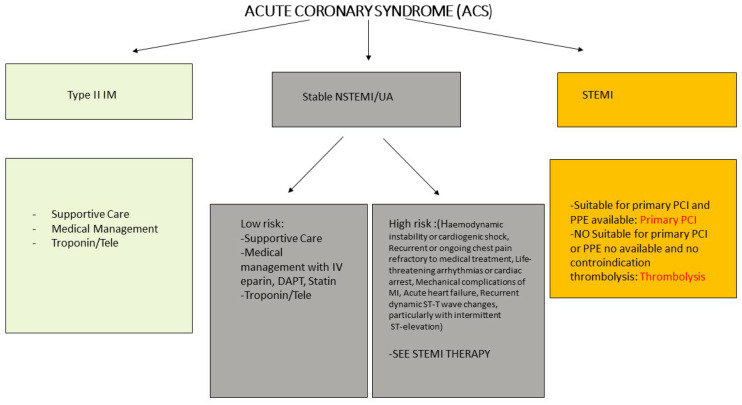
SARS-CoV-2 and ACS management. Type 2 myocardial infarction (type II IM); non-ST segment elevation myocardial infarction (NSTEMI); unstable angina (UA); ST elevation myocardial infarction (STEMI); percutaneous coronary intervention (PCI); dual antiplatelet therapy (DAPT); personal protective equipment (PPE).

**Figure 4:**
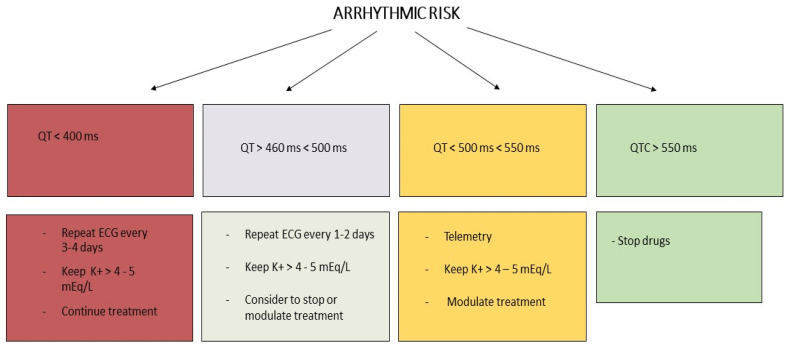
Arrhythmic risk management.

As evidenced by several studies, it seems that the main cause of myocardial damage is myocardial damage in the absence of epicardial coronary artery thrombosis, although it is possible that patients with COVID-19 may still have concomitant acute coronary syndrome. A correct differential diagnosis between the various causes of myocardial injury is therefore essential in patients with myocardial damage, limiting fibrinolysis or coronary angiography to certain cases of ST-elevation myocardial infarction (STEMI) [57,58,59,60] (Figure 2, Figure 3 and Figure 4).

However, one aspect to consider is the high contagiousness of the virus; thus, one of the priorities is to limit the exposure of healthcare personnel to the virus, carrying out only the fundamental tests for diagnosis and management of cardiovascular complications. In fact, in Italy and Spain, 8% and 12%, respectively, of infection are observed in health workers, and although personal protective equipment reduces the risk of contagion, they are unable to cancel it [61].

Another consideration to be made is the cleanliness and hygiene of the operating room and/or the devices used to make a diagnosis that could delay the same tests for other patients who need them.

On the other hand, as a recent Italian study shows, it should also be considered that the high fear of contagiousness and mortality from the virus has led to a drastic decrease in hospitalizations for acute coronary syndrome with a consequent increase in mortality [62].

Finally, another particular group of patients are COVID-19 patients with cancer; they are at high risk of cardiovascular complications due to the association between antineoplastic (for example anthracyclines) or anti-COVID-19 (tocilizumab and ritonavir) drugs and cardiotoxic effects [63,64].

## 7. Conclusions

Several studies have highlighted a clear relationship between COVID-19 infection and the involvement of the cardiovascular system, especially in patients who develop ARDS and those hospitalized in ICUs. However, further studies are needed to clarify the correct management of cardiovascular involvement and any long-term repercussions in the patients involved.

## Figures and Tables

**Table 1:** Studies evaluating cardiovascular involvement during the coronavirus disease (COVID-19) pandemic.

First Author, Year of Publication (Reference)	Country	N° of Patients	Evidence
Poissy J, 2020 [10]	France	107	Pulmonary embolism had a high frequency (20.6%), higher than that observed in influenza patients admitted to the ICU (intensive care unit) for respiratory failure in 2019.
F A Klof, 2020 [11]	The Netherlands	184	Venous thromboembolism confirmed in 27%, arterial thrombotic events in 3.7%.
Lodigiani C, 2020 [12]	Italy	388	Thromboembolic events occurred in 28 (7.7%) patients. Ischemic stroke and ACS (acute coronary syndrome)/MI (myocardial infarction) in 2.5% and 1.1%, respectively. Overt DIC (disseminated intravascular coagulation) present in 8 (2.2%) patients.
Songping Cui, 2020 [13]	China	81	VTE associated with a poor prognosis was present in 25%. The increase in D-dimer identified high-risk groups of VTE.
